# Circularly polarized printed dual port MIMO antenna with polarization diversity optimized by machine learning approach for 5G NR n77/n78 frequency band applications

**DOI:** 10.1038/s41598-023-41302-2

**Published:** 2023-08-26

**Authors:** Ajay Kumar Dwivedi, Nagesh Kallollu Narayanaswamy, Krishna Kanth Varma Penmatsa, Suyash Kumar Singh, Anand Sharma, Vivek Singh

**Affiliations:** 1 Department of Electronics and Communication, Nagarjuna College of Engineering and Technology, Bengaluru, Karnataka India; 2 Department of Electronics and Communication, Sagi Rama Krishnam Raju Engineering College, Bhimavaram, Andhra Pradesh India; 3 Department of Electronics and Communication, Indian Institute of Information Technology Allahabad, Prayagraj, Uttar Pradesh India; 4 Department of Electronics and Communication, Motilal Nehru National Institute of Engineering and Technology Allahabad, Prayagraj, Uttar Pradesh India

**Keywords:** Electrical and electronic engineering, Metamaterials

## Abstract

In this communication, a planar dual port multiple input multiple output antenna of size 1.2λ_0_ × 0.6λ_0_ × 0.008λ_0_ with LHCP/RHCP features is reported for the fifth-generation new radio n77/n78 sub-6 GHz wireless applications band. The single unit of the proposed design consists of a modified L-shape rectangular radiator with Z-shape slot loaded DGS. The defected ground structure is optimized through machine learning algorithms to achieve the maximum ARBW (output) by Right Shifting (RS) and left shifting (LS) the DGS and obtaining input features. The performance metric for ANN with ADAM optimizer was found to be optimal with MSE and R^2^ of 0.99 and 0.82, respectively. ANNs can leverage gradient information to guide the optimization process. This enables faster convergence towards optimal solutions compared to popular GAs and PSO, which are often gradient-free optimization methods. The MIMO configuration is achieved by creating a mirror image of the single unit about the x-axis. The salient features of the proposed design are (a) Impedance bandwidth (IBW) of 3.0–4.2 GHz covering the n77/n78 band, (b) 3-dB axial ratio bandwidth (ARBW) of the 2.6–3.9 GHz (c) Port-1 is generating RHCP while Port-2 is generating LHCP, results in polarization diversity. Different diversity performance parameters (ECC < 0.005, DG ~ 9.99 dB, and MEG < 3 dB) are in the optimum range confirming the proposed configuration as a suitable design for a MIMO radiator.

## Introduction

Due to the increasing number of consumers and the rapid advancement of wireless communication technology, higher throughput, and channel capacity have become crucial needs. Combining several antennas into a single portable device would prove to be a viable option, which will ultimately result in improving the caliber of the communication network and the transmission rate. As a result, the technology known as multi-input, multi-output (MIMO) plays an essential part in the center of activity for 5G research. Throughout the last few years, those working in the telecommunications industry have seen considerable change in the telecom sector. The first generation of mobile communication systems was introduced in (1G). Following the success of 1G, 2G, and 3G, the current standard is 4G. At the moment, 5G is on the verge of becoming a reality. A considerable number of countries have already shown leadership by initiating the rollout of the 5G network. Two potential spectrums might be used for wireless communication using the 5G standard. The first is the range of frequencies below 6 GHz, while the second is the range of microwave frequencies or mm-waves. Since the Sub-6 GHz spectrum is widely accessible in existing wireless communication networks and may be used for 5G without requiring extensive hardware upgrades, service providers are contemplating using this frequency band for the first 5G deployments. The sub-6 GHz band consists of n77/n78/n79: (3.3–4.2)/(3.3–3.8)/(4.4–5.0) GHz^1^. Various performance enhancement methodologies are discussed in the literature to cater the requirements of fifth generation wireless technologies^2–9^. Recent articles in the research literature have described a variety of MIMO antenna designs for 5G networks operating below 6 GHz^10–23^. But compared to their forerunners, these antennas either have a narrower bandwidth or higher mutual coupling. However, the antenna size may grow due to reduced mutual coupling and fewer ECCs between nearby antenna components. Therefore, these factors are crucial in the design of MIMO antennas for handheld electronics. As a result, one of the challenges of antenna design for portable devices is to include several antennas inside the device despite the limited space available while yet ensuring enough isolation. Several techniques are available in the literature to reduce the mutual coupling between the antenna elements in MIMO^24–29^.

On the other side broadband, circularly polarized (CP) antennas have been more in demand in recent years due to the proliferation of high-speed wireless networks. Given its ability to mitigate both polarization mismatch and multipath interference, CP antennas have garnered considerable interest. As per the conceptual definition, a CP is produced by two orthogonal resonant modes with the same amplitude and a 90-degree phase difference. Broadband CP antennas are attractive in several different wireless systems because of the need to support large data rates. In contrast to modern microstrip antennas, conventional microstrip antennas often suffer from narrow bandwidth and a high axial ratio. The challenge of creating a small antenna with a high CP bandwidth has so emerged as a significant area of study. 5G requires small CP antennas. Wireless and satellite applications employ circularly polarized antennas. Due to benefits including suppressing multi-path losses and addressing polarization mismatch of transmitter and reception antennas, CP antenna research is vital.

Wireless networks rely on antenna design. Thus, a simple antenna parameter design and optimization approach is needed. High-frequency simulations take significantly longer as technology reduces antenna size and increases antenna characteristics like frequency and complexity. Machine learning-based models are commonly utilized to overcome these difficulties. These models estimate antenna performance quickly. Antenna design may employ various machine-learning principles and methods^30^. Sharma et al. proposed Lasso (least absolute shrinkage and selection operator), k nearest neighbour, and artificial neural network to improve the double T-shaped monopole antenna^31^. Gao et al. determined the microstrip patch antenna’s resonance frequency using semi-supervised learning-based Gaussian Process Regression (GPR)^32^. Sarkar et al. proposed a band-notch UWB slotted antenna and predicted the notch frequency using a multi-adaptive neuro-fuzzy model^33^. Ranjan et al. built five machine-learning models to propose a UWB-compatible CPW-fed monopole radiator. Predictions match simulations and measurements^34^.

To cater the present fifth-generation requirements of portable wireless communications devices, circularly polarized multiple input and multiple output antennas with high isolation, high gain, and optimum diversity performance are the best solutions. MIMO will provide high channel capacity and data rates with limited power while a circularly polarized feature of the antenna enables it to compensate for the multipath fading, and provide polarization-dependent freedom as compared to the linearly polarized antenna.

In this proposed work, an ML-optimized dual port printed MIMO antenna with wide ARBW is reported for the 5G NR band of n77 (3300–4200 MHz) & n78 (3300–3800 MHz). The mirror image placement of the two antenna elements of the MIMO antenna is producing polarization diversity (Port-1: RHCP, Port 2: LHCP). The antenna has a simple design, and its excitation is provided via a 50-Ω feedline. The design that has been suggested is implemented on a Rogers 5880 laminate that has a thickness of 0.8 mm. The suggested design's main characteristics are as follows: (a) an impedance bandwidth (IBW) of 3.0–4.2 GHz covering the n77/n78 band; (b) a 3-dB axial ratio bandwidth (ARBW) of the 2.6–3.9 GHz; and (c) Polarization diversity as a consequence of Port-1 producing RHCP and Port-2 creating LHCP. The simulated and measured findings agreed well, indicating that the suggested design might be a good contender for 5G communication owing to its low ECC, high DG, and high isolation between the radiators. The results of optimization using machine learning are quite similar to those obtained by simulation and experimentation.

## Antenna designs and geometry

**Figure 1:**
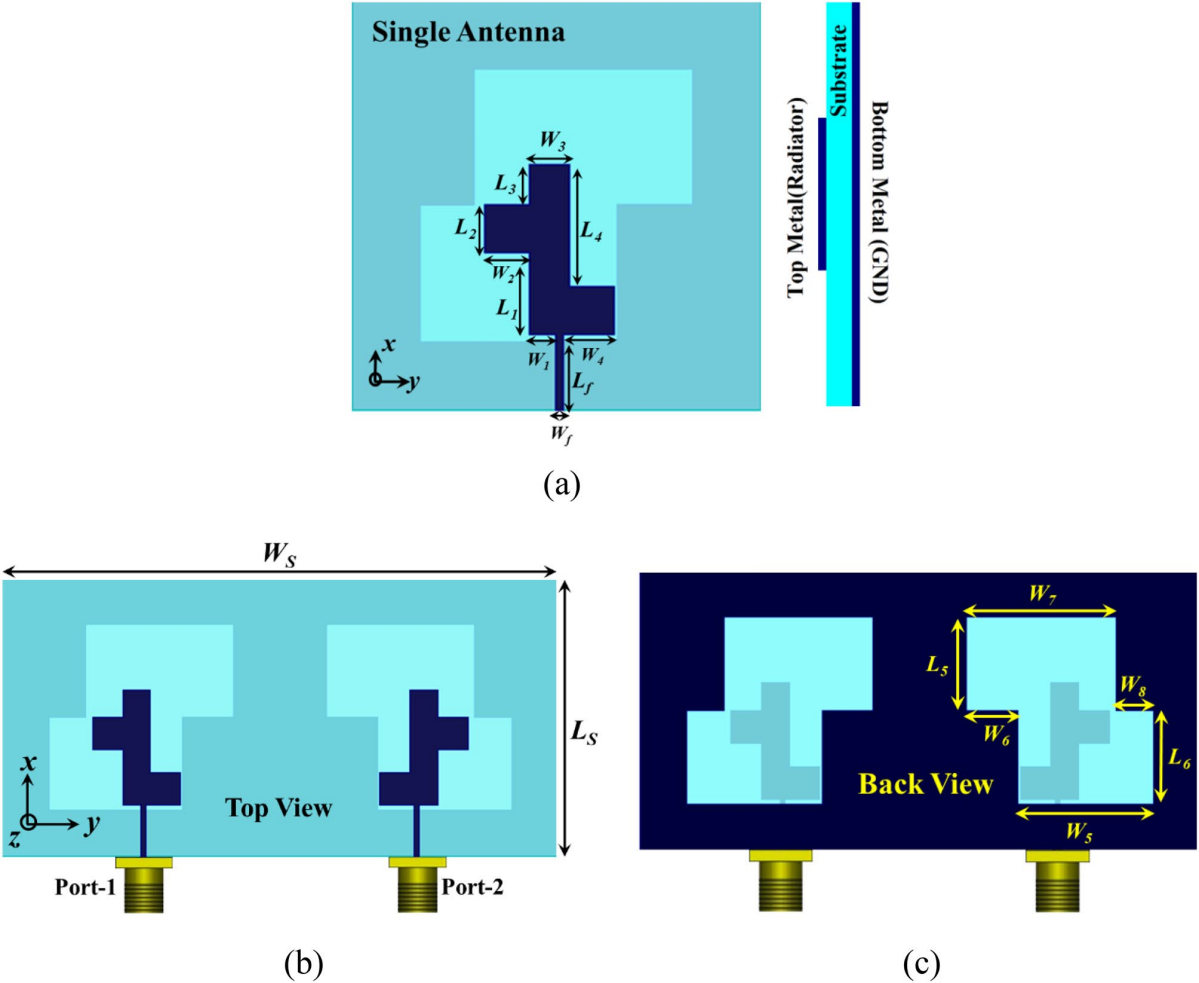
(**a**) Geometrical specification of the unit element of MIMO antenna. Geometrical specification of the MIMO antenna, (**b**) front view, (**c**) back view.

**Figure 2:**
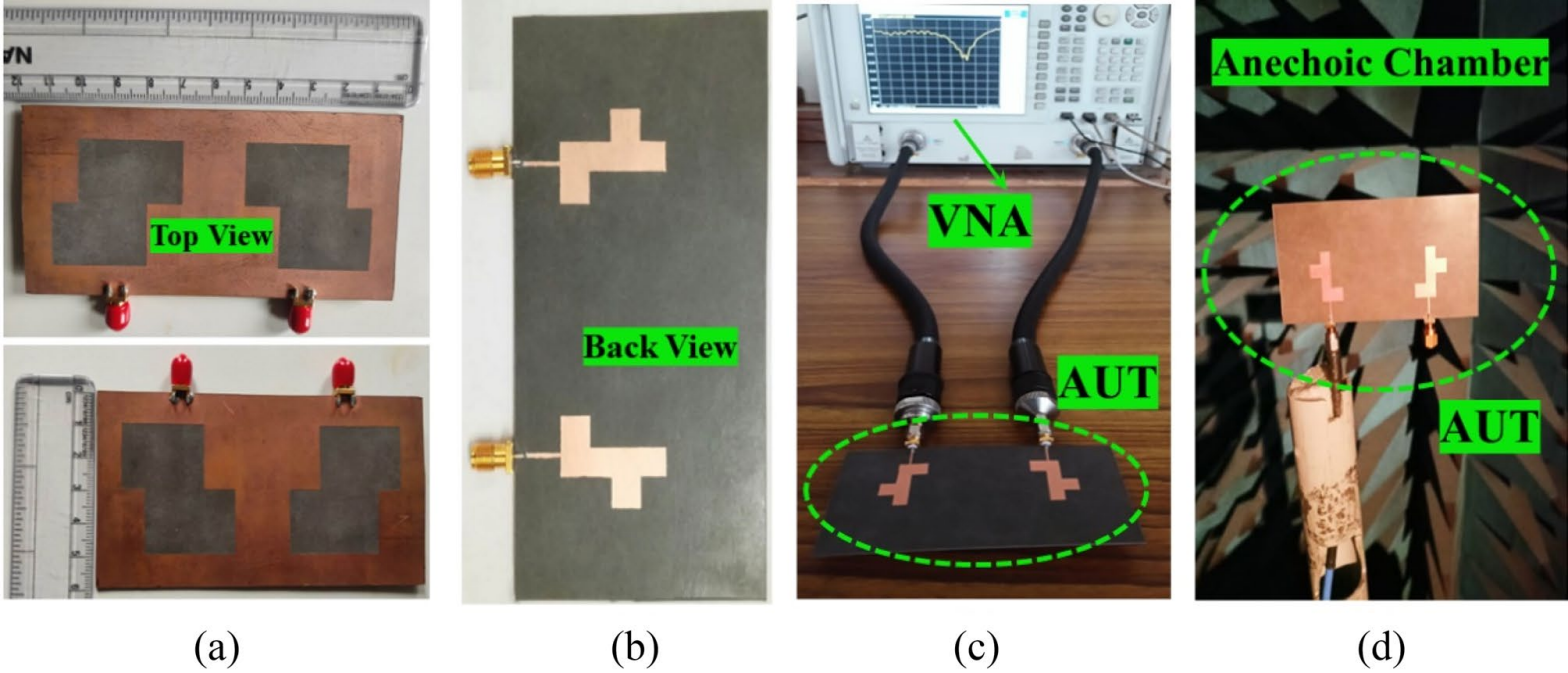
Fabricated and AUT photographs of the proposed design.

The geometrical layout and fabricated photographs of the proposed MIMO antenna are shown in Figs. 1 and 2. Figure 1a shows the front view and side view of a single antenna element while Fig. 1b,c are representing the top view and back view of the proposed MIMO antenna respectively. The fabricated antenna photographs with AUT specifications are shown in Fig. 2. The proposed single antenna element has the total dimensions of 60 × 60 × 0.8 mm^3^, printed on Rogers RT duroid 5880 high-frequency laminates of a thickness of 0.8 mm and relative permittivity of 2.2. The top surface of the substrate consists L-shape radiator with a rectangular stub, fed with a 50 Ω transmission line while the back portion of the substrate is printed with a copper sheet engraved with a Z-shape slot working as the defected ground surface. The suggested MIMO antenna is obtained by replicating the unit cell in mirror orientation about the x-axis which as a result increases the dimension to 120 × 60 × 0.8 mm^3^.

The dimensional specifications of the proposed antenna with geometrical parameters are mentioned as: L_1_ = 12 mm, L_2_ = 7 mm, L_3_ = 6 mm, L_4_ = 18 mm, L_5_ = 20 mm, L_6_ = 20 mm, L_f_ = 11 mm, L_s_ = 60 mm, W_1_ = 4 mm, W_2_ = 6.5 mm, W_3_ = 6 mm, W_4_ = 7.5 mm, W_5_ = 29 mm, W_6_ = 8 mm, W_7_ = 32 mm, W_8_ = 11 mm, W_f_ = 1 mm, W_s_ = 120 mm, thickness of substrate = 0.8 mm.

## Single antenna analysis

### Antenna design evolution stages

**Figure 3:**
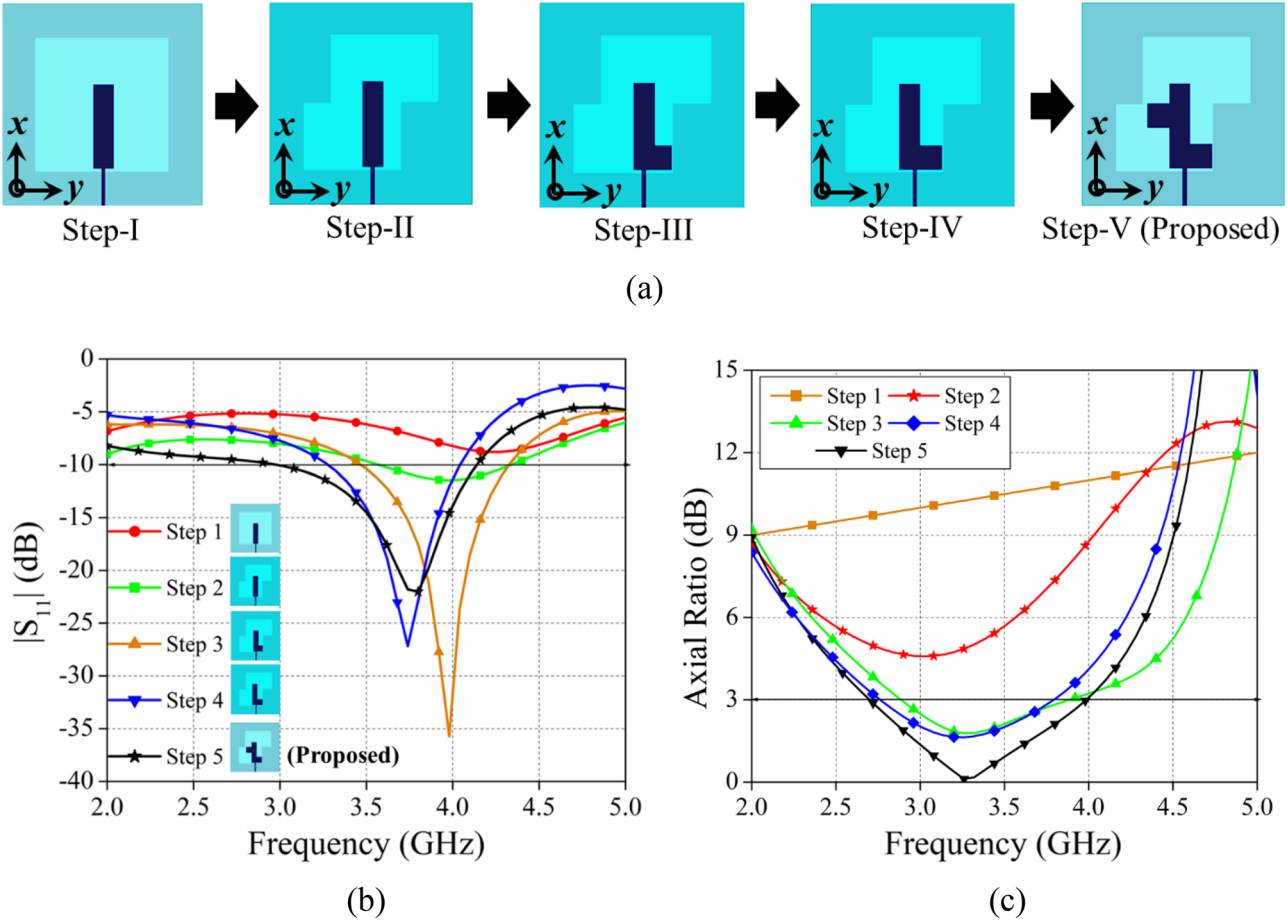
(**a**) The evolution steps for the proposed single antenna element. (**b**) |S_11_|, (**c**) Axial ratio, plot for the different antenna evolution steps.

The different evolution stages of the proposed single antenna element are discussed and investigated in this section. To achieve the final design of the proposed antenna five different steps (step-1 to step-5) are presented and a comparative investigation is carried out in terms of |S_11_| (dB) and Axial ratio (dB) (cf. Figure 3). In step-1, a simple rectangular radiator with a square ring shape defected ground surface is designed; resulting in single resonance at 4 GHz without CP. In step-2, the square slot in the ground is converted into a Z-shape slot to increase the impedance bandwidth and to achieve the CP. In step-3, the rectangular radiator is converted into an L-shape radiator resulting in resonance at 3.8 GHz with wider impedance bandwidth and 1 GHz of 3-dB ARBW. Further, the positional variation of feed location is carried out to find out the optimum value of impedance bandwidth and 3-dB axial ratio bandwidth result in step-4. Even though step-4's 3 dB impedance bandwidth is sufficient to cover the 5G NR Bands n77 and n78, we explore alternative ways to increase impedance matching and axial ratio over the target band as much as possible, which leads to the creation of quarter wavelength stub in the radiator originated the step-5 increasing 3-dB axial ratio bandwidth of 2.6–3.9 GHz with maximum impedance bandwidth of 3–4.2 GHz.

### Parametric investigation

**Figure 4:**
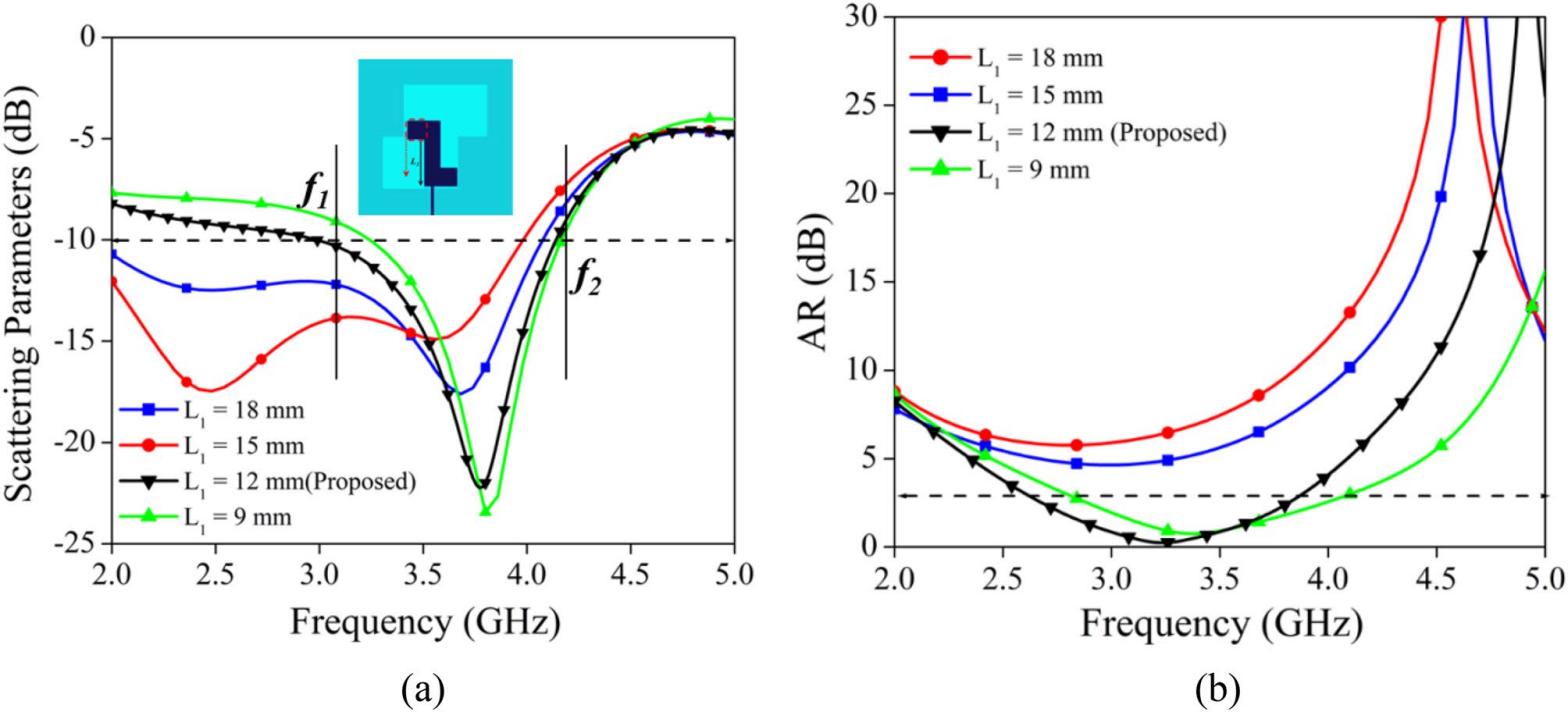
Parametric analysis in terms of different values of L_1_ (**a**) |S_11_|, (**b**) |AR|.

To investigate the effect of different parameters dimension on the impedance bandwidth and axial ratio, a parametric investigation is carried out in terms of quarter wavelength stub position (L_1_), and feed position (W_1_). The quarter wavelength stub is playing important role in getting the wider axial ratio bandwidth. From the perusal of Fig. 4a,b it is observed that the positional variation of the stub from L = 18 mm to L = 9 mm is significantly affecting the impedance bandwidth and ARBW. As the position of the stub is shifting downward, the lower cutoff frequency (f_1_) is moving towards the higher frequency range, and a small shifting effect is observed for higher cutoff frequency (cf. Figure 4a). While the downward movement of the stub from L = 18 mm to L = 12 mm caused the enhancement of 3-dB ARBW. Further shifting of the stub from L = 12 mm to L = 9 mm resulted in a decrease in the ARBW. The optimum value of impedance bandwidth and ARBW is obtained at L = 12 mm.

**Figure 5:**
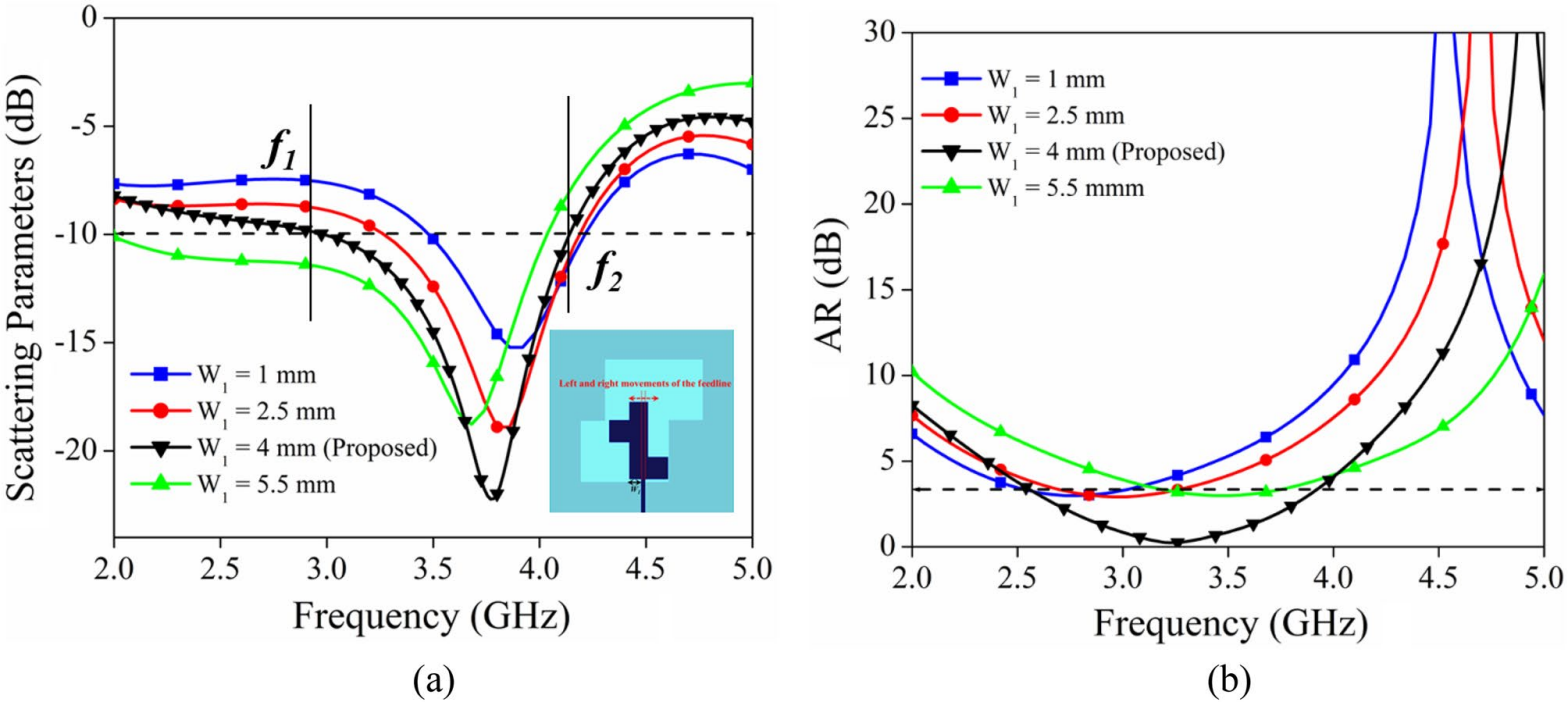
Parametric analysis in terms of different values of W_1_ (**a**) |S_11_|, (**b**) |AR|.

The variation of |S_11_| and 3-dB ARBW for feed position (W_1_) shifting is presented in Fig. 5a,b respectively. From the observation of Fig. 5a, it is clear that the positional shifting of the feed from W_1_ = 1 mm to W_1_ = 5.5 mm result in a shift of the lower cutoff frequency (f_1_) in the lower frequency range while marginal shifting is observed in case of the higher cutoff frequency. Further, in the case of 3-dB ARBW variation, the optimum value is achieved in the case of W_1_ = 4 mm only (cf. Figure 5b). The maximum value of ARBW with desired bandwidth for n77 (3300–4200 MHz) & n78 (3300–3800 MHz) is obtained for W_1_ = 4 mm.

### Optimization to achieve CP through ML Algorithms

**Figure 6:**
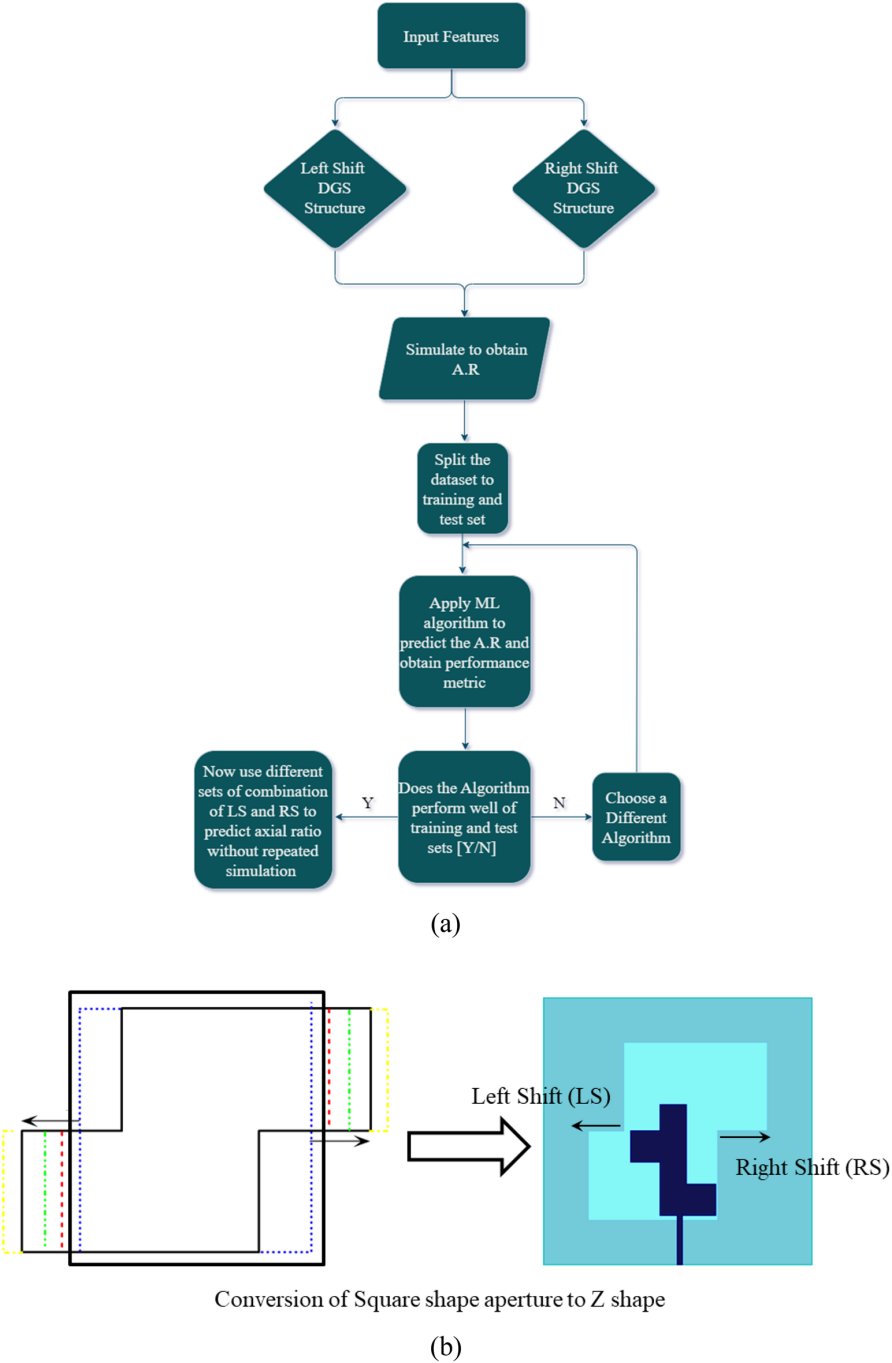
(**a**) Flow chart of the proposed machine learning optimization algorithm. (**b**) Right shift (RS) and Left shift (LS) of DGS structure in the proposed antenna.

In order to understand the implementation of machine algorithm in antenna axial ratio bandwidth optimization, a flowchart is incorporated in the Fig. 6a. To predict the axial ratio for better optimization and attainment of CP for the proposed antenna, different ML- algorithms were incorporated in this study. Since the goal is to exploit ML algorithms that not only provide optimized predicted outcomes but also can be generalized to different types of antennae as well. However, since many ML algorithms such as polynomial regression, support vector regression, decision tree, etc. suffer from overfit of the datasets, therefore, at first a type of gradient boosting algorithm i.e., CAT Boost is incorporated which restrict the overfitting by incorporating early stopping round parameters and hence solve the problem of complex overfitting.

Further, an artificial neural network (ANN) is incorporated to improve the predicted outcome. The independent parameters X_i_ are obtained from the right shift (RS) and left shift (LS) of the Diffracted ground structure as shown in Fig. 6b. The dependent parameter Y_i_ is the axial ratio obtained from different combinations (in the range of − 2 mm to + 2 mm) of spatial movement of the DGS structure as shown in Table 1 (only 10 percent of the total datasets is shown here).

**Table 1:** Independent variable (LS and RS) and dependent variable (axial ratio).

Right shift (RS)	Left shift (LS)	Axial ratio in dB
− 2	2	7.9
− 1.5	2	8.9
− 1	2	7.94
− 0.5	2	10.08
0	2	10.9
0.5	2	12.7
1	2	14.76
1.5	2	2.38
− 2	− 2	1.71
− 1.5	− 2	1.13

### CATBoost algorithm

The objective function in CATBoost is defined as follows^35^:

1 $$ Loss\left( {y,\mathop y\limits^{ \wedge } } \right) = \sum\limits_{i = 1}^{n} {L\left( {y_{i} ,\mathop {y_{i} }\limits^{ \wedge } } \right)} + \sum\limits_{i = 1}^{T} {\Omega \left( {f_{i} } \right)} $$

where y is the test target value, ŷ is the predicted target value, L is the loss function, T is the number of trees in the model, ${\text{f}}_{{\text{i}}}$ is the prediction of the ith tree, and Ω is the regularization term. The loss function L can be one of several different options, such as the mean squared error (MSE) for regression problems or the cross-entropy loss for classification problems. The regularization term Ω serves to penalize the complexity of the trees in the model, helping to prevent overfitting. The specific form of Ω depends on the type of regularization being used. For example, the “leaf values regularization” term is defined as:

2 $$ \Omega (f) = \lambda \sum\limits_{j = 1}^{LeafCount} {w_{j}^{2} } $$

where λ is a regularization coefficient, Leaf Count is the number of leaves in the tree, and ${\text{w}}_{{\text{j}}}$ is the average value of the target in the j-th leaf. Overall, the objective function in CAT Boost is designed to minimize the loss on the training data while also regularizing the model to prevent overfitting.

**Figure 7:**
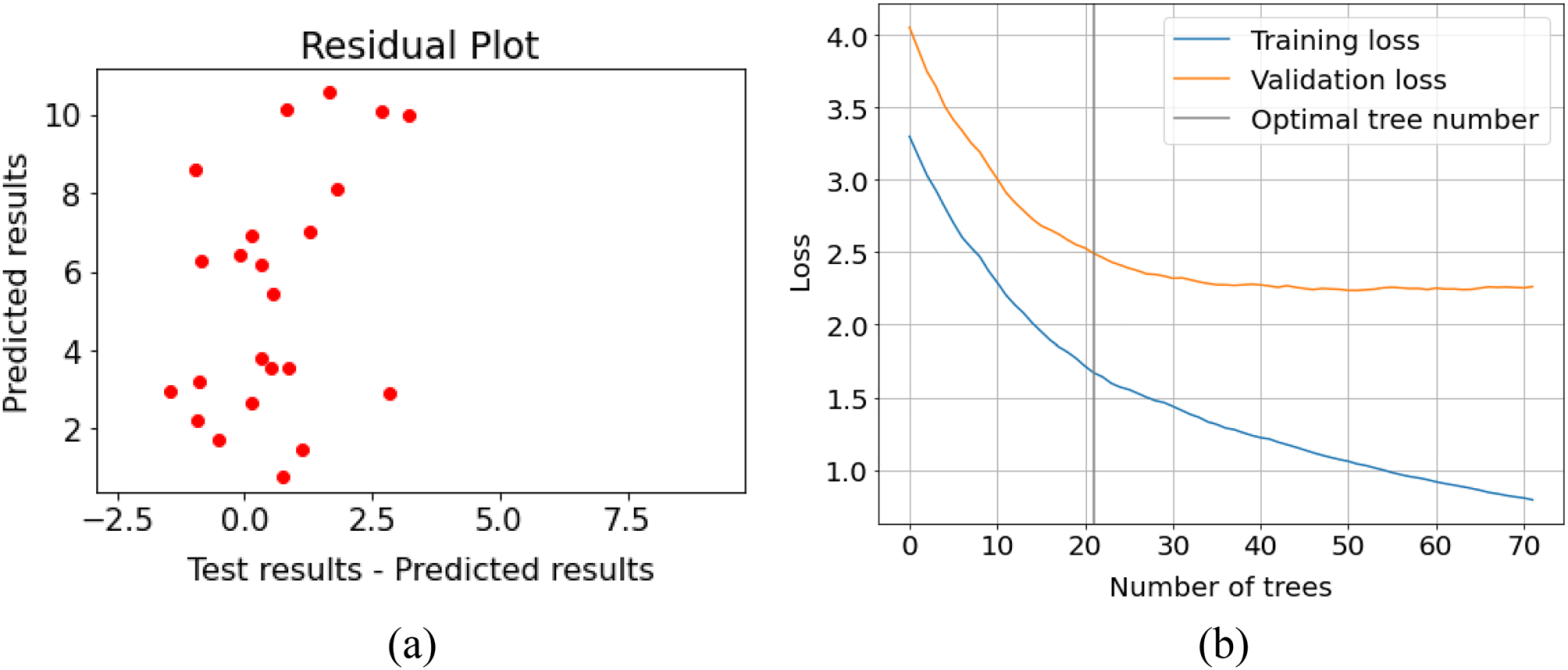
(**a**) Residual plot for CATBoost with a maximum deviation of 2.6 dB, (**b**) Model loss function plot with early stopping round of 20.

The test value in the proposed methodology is the axial ratio calculated for each Right Shift (RS) and Left Shift (LS) of the defected ground structure (DGS) as shown in Table 1. As shown in Fig. 7a the residuals (Test results- Predicted results) are in the range of − 1.7 to 2.6 dB which is acceptable. Further, since the gradient boost method (GBM) may suffer from overfitting of the predicted results, therefore, an early stopping parameter with an optimal tree number of 20 is utilized to improve the efficacy of the algorithm as shown in Fig. 7b. Also, the best-fit MSE (mean squared error) is 2.52, and the R^2^ value of 0.73 indicates that the model fits the data satisfactorily.

## ANN optimization

Since the axial ratio of the antenna is dependent on the right and left shift of the DGS, therefore, for optimized axial ratio and to improve the CP performance of the antenna, the ANN algorithm is further incorporated in this work. Since ANN is unaffected by overfitting, it can be a better substitute for GBM where overfitting is constrained by using an early stopping parameter. Such a parameter improves overfitting but restricts the potential of GBM. Therefore, ANN with three different optimizers is further exploited to improve the predicted outcome.

### Stochastic gradient descent (SGD)

**Figure 8:**
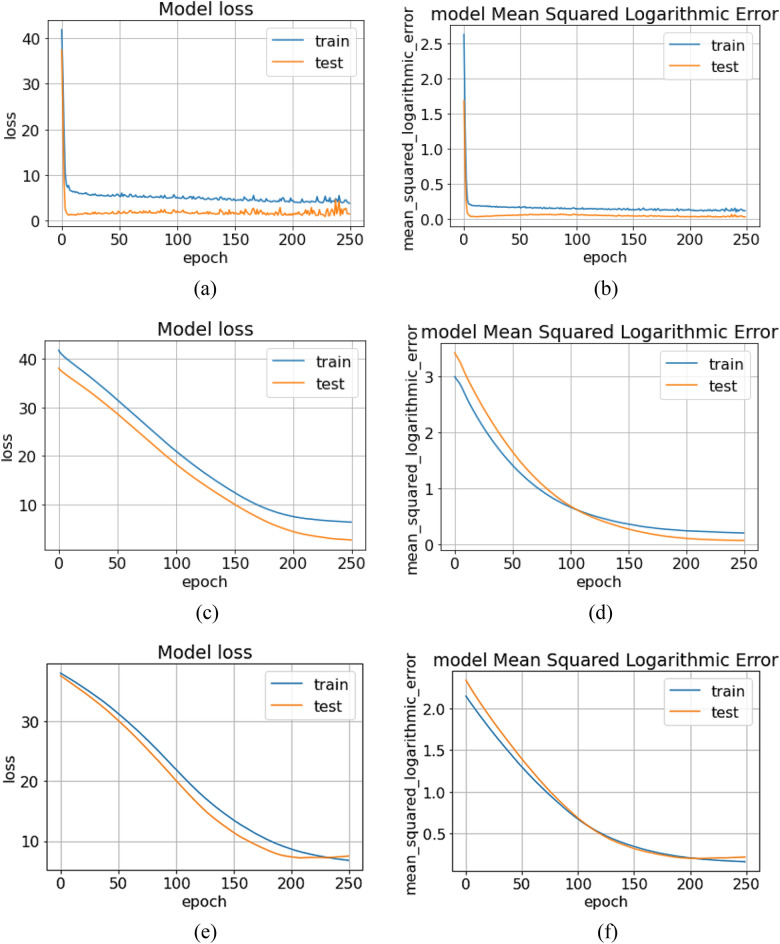
(**a**, **b**) Plot for Model Loss and mean square logarithmic error, respectively for SGD with epoch of 250. (**c**, **d**) Plot for Model Loss and mean square logarithmic error, respectively for RMSprop. (**e**, **f**) Plot for Model Loss and mean square logarithmic error, respectively for ADAM.

Stochastic gradient descent (SGD) is a popular optimization algorithm for training machine learning models, particularly in cases where computing the gradient of the loss function concerning the model parameters is computationally expensive. Instead of computing the gradient based on the entire dataset, which can be time-consuming and memory-intensive, SGD uses a single example (or a small, randomly-selected subset of examples) to compute the gradient, which makes the algorithm much faster. The stochasticity in the name refers to the fact that the algorithm uses randomness in the data to compute the gradient, rather than the full data set. In SGD the regularization hyperparameter θ updated by^36^:

3 $$ \theta = \theta - \alpha \nabla_{\theta } J\left( {\theta ;x^{\left( i \right)} y^{\left( i \right)} } \right) $$

where ${\upalpha }$ is the learning rate and (${\text{x}}^{{\left( {\text{i}} \right)}} ,{\text{y}}^{{\left( {\text{i}} \right)}} )$ are pair of training sets. As shown in Fig. 8a,b, for training set ${\text{x}}^{{\left( {\text{i}} \right)}} ,{\text{y}}^{{\left( {\text{i}} \right)}}$ (Right Shift (RS) and Left Shift (LS)), the predicted outcome (axial ratio) the model loss converges to a minimum with a maximum epoch of 250. However, the model suffers from oscillation which may result in an unstable prediction of the outcome. Therefore, in order to eliminate the oscillation in the model RMSprop optimizer is further incorporated.

### Root mean squared propagation (RMSprop)

Root Mean Squared Propagation (RMSprop) is a gradient descent optimization algorithm used in machine learning. It is similar to the more popular stochastic gradient descent (SGD) algorithm, but with a different approach to the learning rate. In RMSprop, the learning rate is adaptively adjusted for each weight, rather than being kept constant as in SGD. The main idea behind RMSprop is to divide the learning rate for a weight ${\text{w}}_{{\text{t}}} { }$ by a running average of the magnitudes of the recent gradients for that weight. This helps to eliminate the oscillations that can occur when the learning rate is high, and also makes the algorithm more sensitive to changes in the gradients. The first order weight is given by^37^:

4 $$ w{}_{t + 1} = w_{t} - \frac{{\alpha_{t} }}{{\left( {v_{t} + \varepsilon } \right)^{1/2} }}\left[ {\frac{\delta L}{{\delta w_{t} }}} \right] $$

${\text{w}}_{{\text{t}}}$ is time-dependent rate function, the Loss Function derivative is ${\updelta }$ L, the learning rate is ${\upalpha }_{{\text{t}}}$, the sum of the square of past gradients is ${\text{v}}_{{\text{t}}}$. As seen in Fig. 8c,d. The oscillation in the model loss is dampened as compared to a model loss of SGD with a similar Mean Squared error and R^2^ value with the same ratio of the training set and test sets. Further, it can be conferred that the RMSprop algorithm performed better in the prediction of axial ratio as compared to the SGD algorithm.

### Adaptive moment estimation ADAM

It is common practice to use an optimization technique like ADAM (Adaptive Moment Estimation) while instructing a neural network. The technique employs moving averages of the parameters to continuously estimate the second raw moments of the gradients, and the word adaptive in the name alludes to the fact that the algorithm adjusts the learning rates of each parameter depending on the previous gradient information. The equation for ADAM is given by^38^:

5 $$ m_{t} = \beta_{1} m_{t - 1} + (1 - \beta_{1} )\frac{\delta L}{{\delta w_{t} }}v_{t} = \beta_{2} v_{t - 1} + (1 - \beta_{2} )\left[ {\frac{\delta L}{{\delta w_{t} }}} \right]^{2} $$

${\text{m}}_{{\text{t}}}$ is time dependent aggregate of gradients (initially 0), ${\text{m}}_{{{\text{t}} - 1}}$ is aggregate of gradients at a previous value, and the moving average parameter β. As seen in Fig. 8e,f. The Mean Squared error and R^2^ value with a ratio of the training set and test sets of 80:20 are better than SGD and RMSprop. Further, it can be conferred that the ADAM algorithm performed better in the prediction of axial ratio as compared to other ANN optimizers.

Further, the performance parameter of each of the algorithms is shown in Table 2. It can be conferred that the performance of SGD and RMSprop was similar but with the added advantage of RMSprop in suppressing the model oscillation which further facilitates the stability and accuracy of the model.

**Table 2:** Performance parameter of different ANN optimizer.

Parameters	Value (SGD) 80:20	Value (RMSProp) 90:10	Value (ADAM) 90:10
Max error	4.12	4.72	1.95
Mean square error	1.995	2.38	0.99
R^2^ score	0.75	0.73	0.82
Mean squared log error	0.11	0.13	0.07

**Figure 9:**
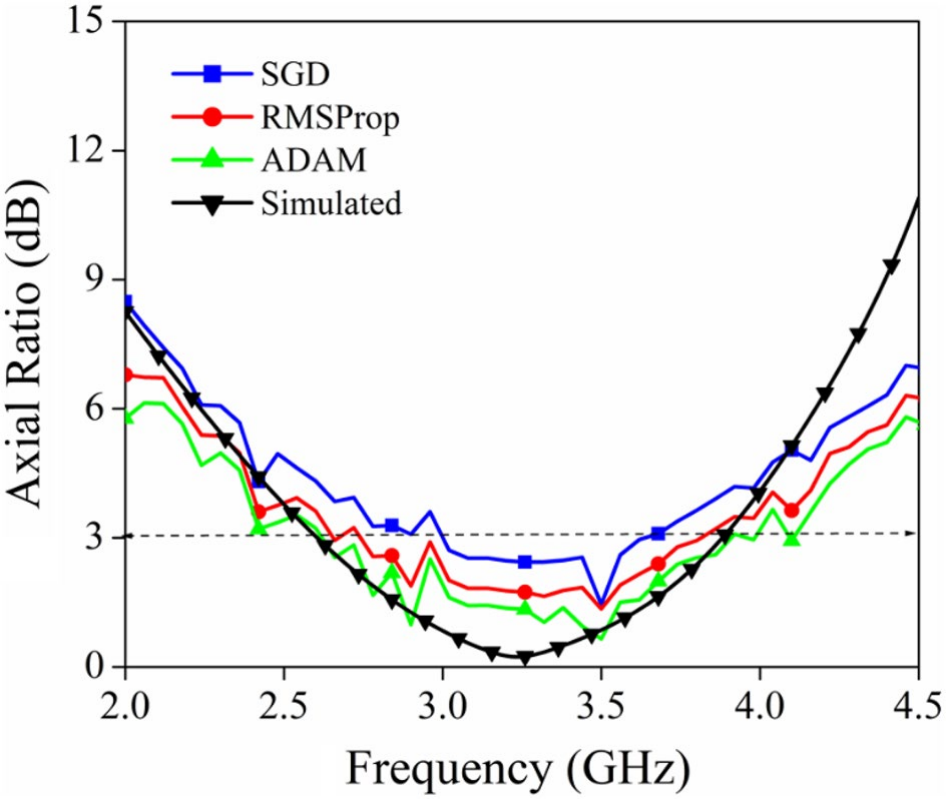
Plot for axial ratio and frequency for simulated and predicted outcomes with 3 ANN optimizers.

Figure 9 shows the simulated and predicted axial ratio in the frequency range of 2–4.5 GHz. As can be inferred, the prediction of the axial ratio by ANN with ADAM optimizer is optimal and thus can be further incorporated in the prediction of CP using different spatial changes in DGS.

### Surface current distribution and CP mechanism

**Figure 10:**
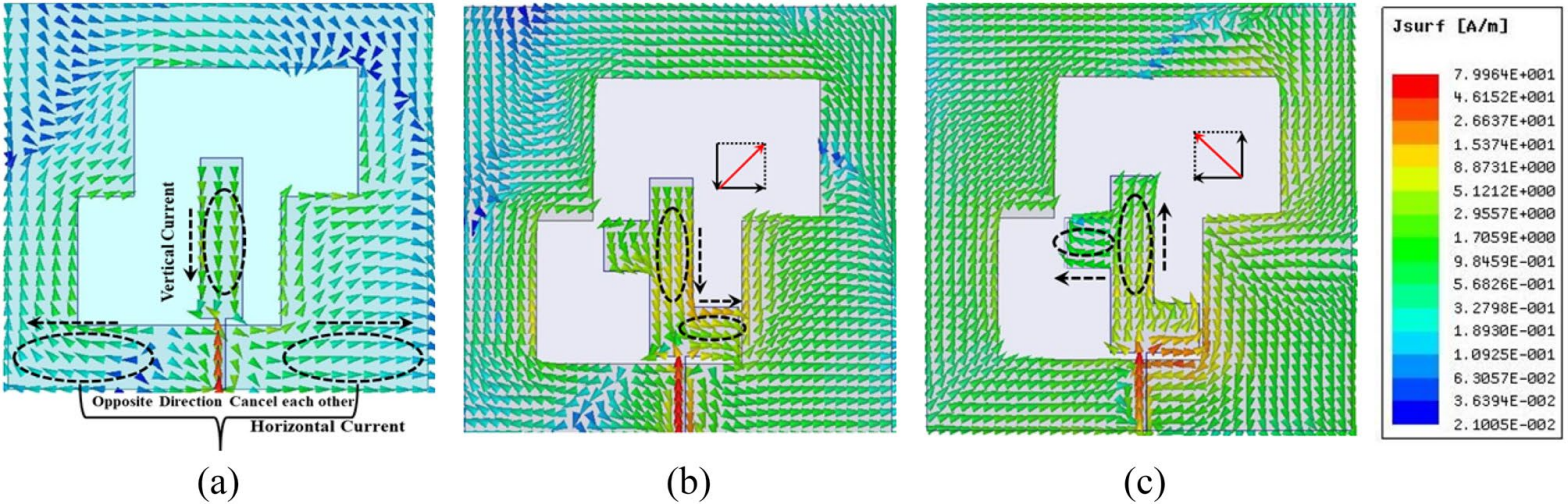
Simulated surface current distribution (**a**) without stub, (**b**) at lower stub, (**c**) at upper stub.

To generate circular polarisation, two electric field components (horizontal (E_X_) or vertical (E_Y_)) must have the same amplitude but be rotated by 90 degrees concerning one another. Figure 10a depicts the surface current distribution of the suggested model which does not include any quarter wavelength stubs. Figure 10a makes it clear that the components of the horizontal surface current in the ground plane are moving in the opposite direction. This may be observed by examining the diagram. Since of this, in the case of the far-field situation, the horizontal radiation is essentially non-existent because its potency is completely nullified. On the other hand, as shown in Fig. 10b,c, the positioning of asymmetric quarter wavelength stubs around the feed line results in the production of two orthogonal currents: one horizontal (E_X_), and one vertical (E_Y_). Strong E_X_ and E_Y_ components are formed as a direct result of the currents that are generated on the stubs. Because the quarter wavelength stubs are oriented asymmetrically, the ground plane's current distributions are likewise altered simultaneously with this process. In the suggested radiator, a quarter wavelength stub functions as an electric dipole while an inverted Z-shape slot loaded ground plane acts as a magnetic dipole, creating orthogonal electric field components. Quarter annular stub adds λ/4 to microstrip route latency. This extra route delay creates a CP wave at 3.5 GHz by shifting the phase of orthogonal electric field lines by 90 degrees (phase difference = 2π/λ × path difference)^39^.

### CP handedness

**Figure 11:**
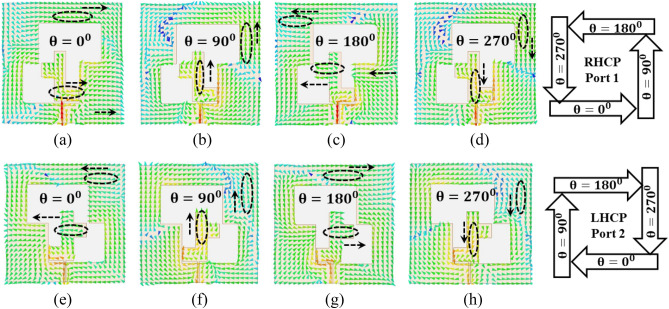
Surface current distribution to show the CP handedness at 3.5 GHz (**a**) Port-1, (**b**) Port-2.

The handedness of the CP wave is explained in Fig. 11, which also incorporates pertinent observations into its depiction of the surface current distribution of the proposed antenna. The graphs of the current distribution are provided for various values of theta (0 degrees, 90 degrees, 180 degrees, and 270 degrees) at a frequency of 3.5 GHz. It is suggested that the right-handed circular polarization (RHCP) is present due to the rotation of surface currents in an anti-clockwise direction at port-1 at the operating frequencies while left-handed circular polarization (LHCP) is present due to the rotation of surface currents in a clockwise direction at port-2 at the operating frequencies.

**Figure 12:**
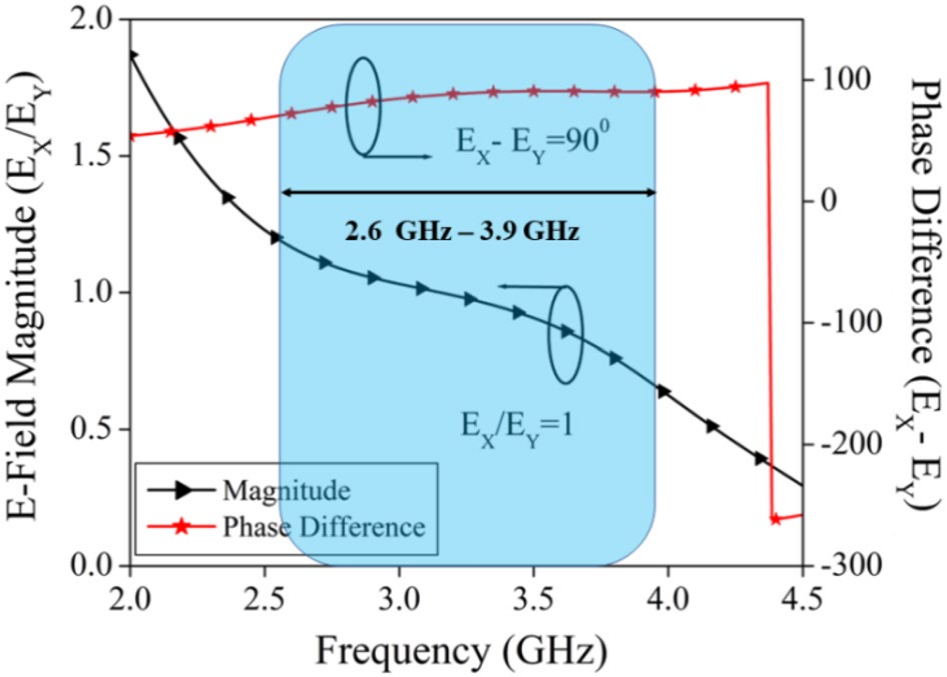
Magnitude and phase diagram for CP mechanism.

Figure 12 shows a graph of the ratio of the size of the horizontal and vertical electric field components (E_X_/Ey) and their phase difference (E_X_-Ey) to help you better understand the proposed antenna's circular polarization. For the impedance bandwidth of 3–4.2 GHz that was found, the ratio of field components is close to 1 and the phase difference is about 90°, which explains the planned antenna has a large circular polarization bandwidth.

## MIMO antenna analysis

The MIMO configuration under consideration is achieved through the replication of the antenna element of the unit cell in a mirror image pattern along the x-axis. In the context of MIMO designs, the grounding sections of individual elements were interconnected to maintain uniform voltage levels across the entire antenna system.

**Figure 13:**
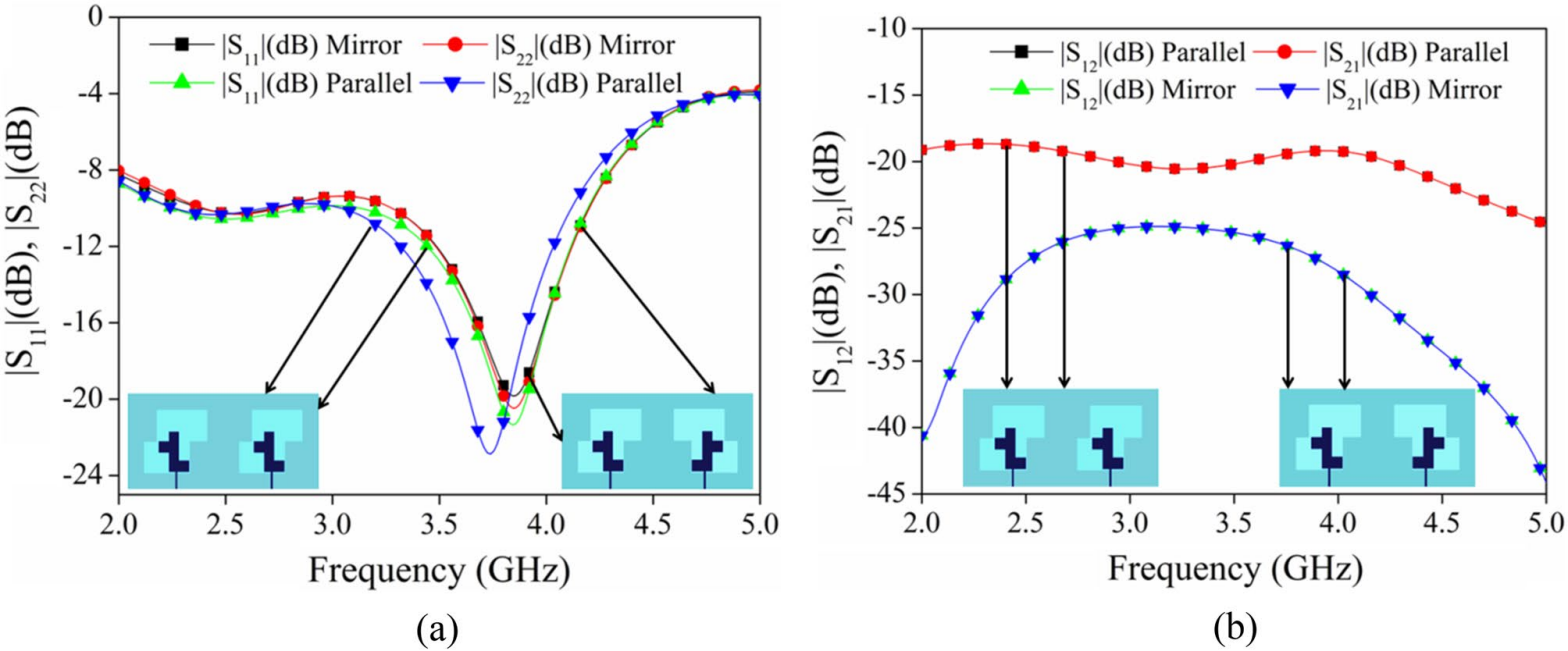
Scattering parameters of the MIMO antenna in Parallel and Mirror configurations (**a**) |S_11_|, |S_22_|, (**b**) |S_12_|, |S_21_|.

Figure 13a,b compare the MIMO antenna return loss and isolation, produced by repeating a single antenna element in parallel and mirror configurations. From the perusal of Fig. 13a, it is observed that the |S_11_| and |S_22_| plots are not in coherence for parallel orientation as compared with the mirror orientation. Similarly from Fig. 13b, the isolation between the antenna elements in parallel orientation is more than 20 dB while for mirror orientation it is more than 25 dB. From the aforementioned discussion, it is clear that the mirror positional placement of the antenna elements is optimum compared with the parallel placement of antenna elements.

**Figure 14:**
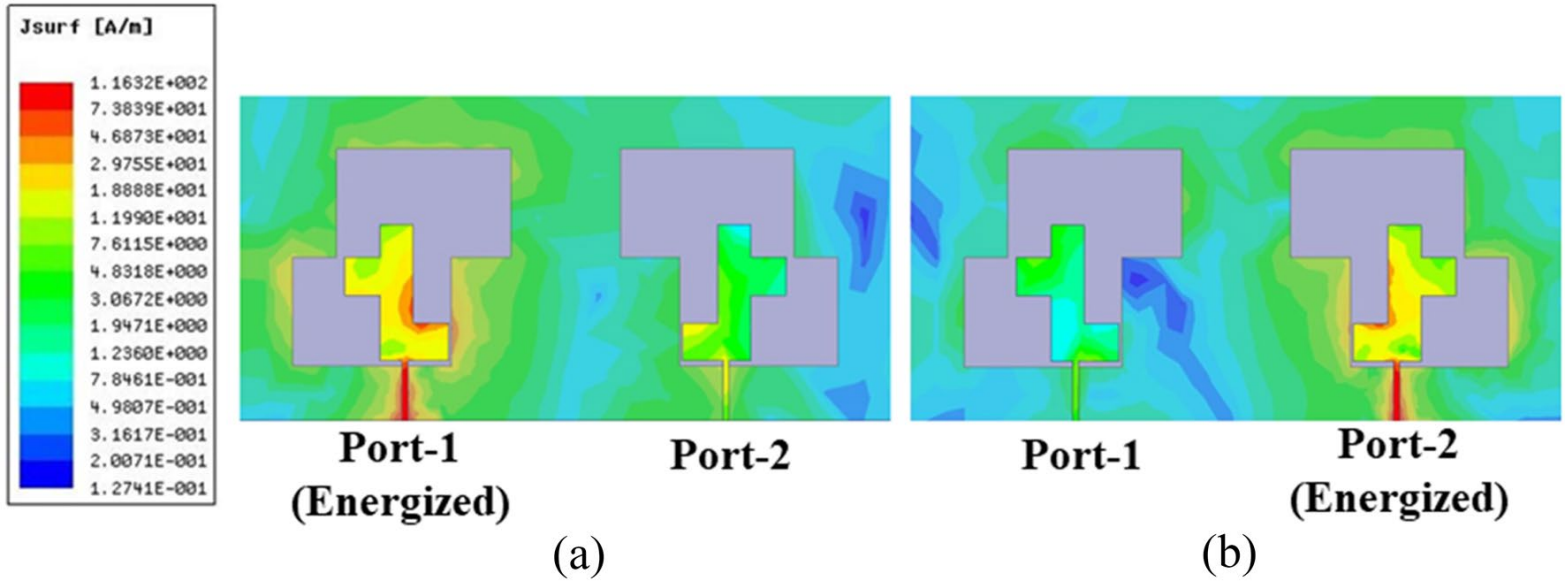
Surface current density for (**a**) Port-1 energized, (**b**) Port-2 energized.

Figure 14 shows the surface current intensity distribution at each dual-port MIMO antenna port. The radiating source has the highest current. This suggests that structural components are the main contributors to resonance within the targeted frequency range. For each antenna element, the feed line has some electric field strength. A mirror design minimizes coupling fields between antenna components. Figure 14a shows the current density when port-1 is stimulated, whereas Fig. 14b shows port-2.

## Results and discussion

**Figure 15:**
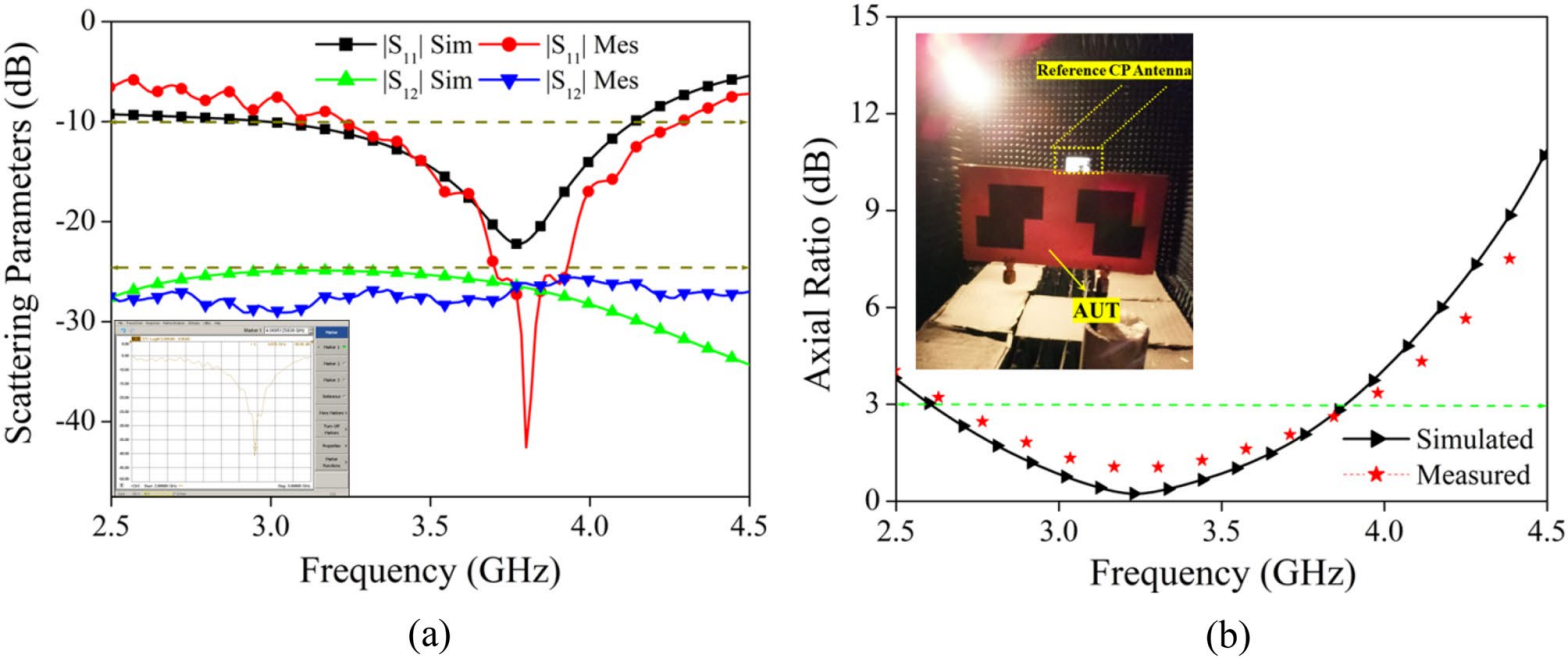
Simulated and measured (**a**) scattering parameters, (**b**) axial ratio of the Proposed design.

This section focuses on three key points: (i) comparing simulated and observed findings (ii) calculating diversity performance (iii) examining how well the proposed two-port MIMO antenna stacks up against the other antennas that have been published in the literature. Figure 2 shows the antenna prototype being tested using the Vector Network Analyzer (VNA) to validate simulated performance. Figure 15a,b demonstrate the proposed MIMO antenna measured and simulated |S_11_|, |S_12_| and axial ratio variations. By looking at Fig. 15a, it can be noted that the suggested antenna design has a resonant band from 3.0 GHz to 4.2 GHz (simulated), 3.17 GHz to 4.55 GHz (Measured) with CP responses from 2.6 GHz to 3.9 GHz (Simulated), 2.66 GHz to 3.92 GHz (Measured). The axial ratio is determined by measuring the pattern in a Dual-Linear fashion. The axial ratio of the proposed antenna may be determined by performing the following steps^40^:

i. The initial procedure involves orienting the proposed test antenna in the XZ plane, while maintaining a polar angle of θ = 0° and an azimuthal angle of ϕ = 0°.
ii. Determine the desired frequency interval for measuring the axial ratio.
iii. At this juncture, it is recommended to rotate the horn antenna from its initial position of 0° to a full 360° rotation in order to attain co-polarization. Take down the dBm readings from the spectrum analyzer showing how much power the test antenna was able to receive at each angle.
iv. Since circular polarization is a subset of elliptical polarization, it is clear that the two are related. Now the main axis represents the highest power level and the minor axis represents the lowest. Since power is measured in decibels (dB), you may calculate it by subtracting the lowest power (major axis) from the greatest power (minor axis). Subtracting two numbers yields the axial ratio.

Figure 15 displays the simulation and measurement results, which show that the inclusion of the SMA connections and the inevitable measurement errors result in modest variations in the S-parameters and axial ratio.

### Gain and radiation efficiency

**Figure 16:**
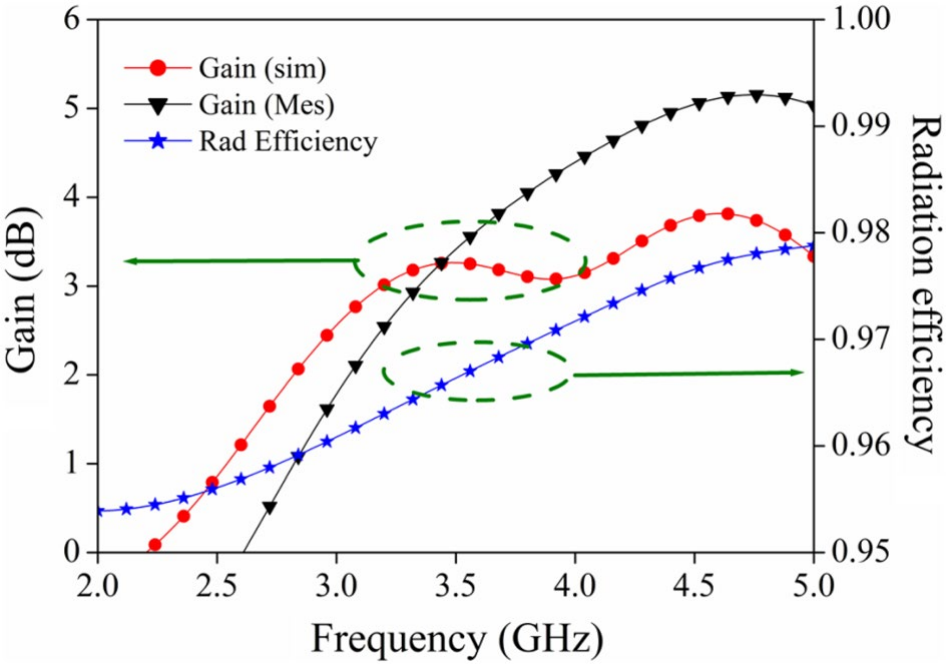
Simulated/measured antenna gain and radiation efficiency.

Figure 16 illustrates the gain (both measured and simulated) as well as the radiation efficiency plot of the MIMO antenna. An overall gain of 2–4 dB is achieved over the CP band, with a peak gain of 4 dB at 4 GHz. From the perusal of this plot, two conclusions can be implied: (a) an increase in gain is seen with an increase in frequency because the effective aperture of an antenna is larger with respect to the wavelength; (b) the radiation efficiency is more than 95% over the whole impedance bandwidth.

In order to measure the gain of proposed antenna, Two Antenna Method is used [B]. The steps involved in the measurement of antenna gain are as follow:

i. The proposed antenna is mounted in broadside direction.
ii. Standard Gain antenna on the other side (whose gain is known to us i.e. GT)
iii. Now find out S21 at each frequency points which give (PR-PT).
iv. Path losses are finding out by $20{ }\log_{10} \left( {\frac{{4{\pi R}}}{\uplambda }} \right)$; R is the distance between Transmitting and receiving antenna
v. Find out cable loss by directly connecting the transmitting and receiving cable.

Now, finally used Friss Equation formula given as follow:

$$ {\text{G}}_{{\text{R}}} = {\text{S}}_{21} \left( {{\text{in}}\;{\text{dB}}} \right) + 20\;\log_{10} \left( {\frac{{4{\pi R}}}{\uplambda }} \right) - {\text{G}}_{{\text{T}}} + {\text{cable}}\;{\text{loss}}{.} $$

### Radiation pattern

**Figure 17:**
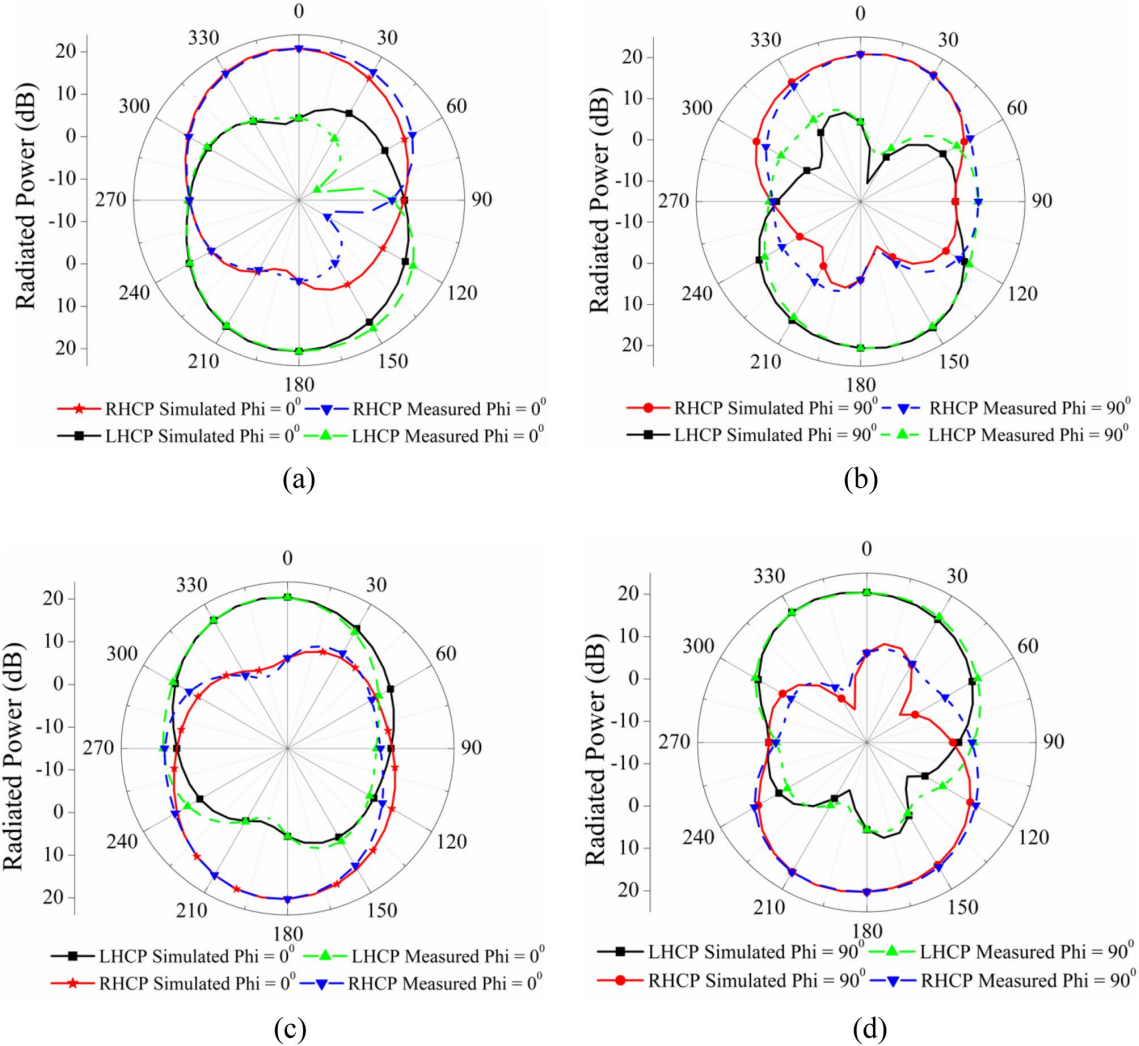
Simulated and measured LHCP/RHCP radiation pattern at 3.5 GHz (**a**) Port-1 in $xoz$ plane, (**b**) Port-1 in $yoz$ plane, (**c**) Port-2 in $xoz$ plane, (**d**) Port-2 in $yoz$ plane.

The suggested antenna’s circular polarization plots for ports 1 and 2 in left-handed (LHCP) and right-handed (RHCP) variants are shown in Fig. 17. One can obtain an estimation of the radiation pattern through the utilization of a matching load termination on a port, while simultaneously offering excitation to other ports. First of all, measure the radiation pattern of test antenna in two orthogonal planes by placing reference antenna (horn antenna) in E-and H-plane respectively. By doing so, we get E_H_ and E_V_ value for different angle at specific frequency. Now, LHCP and RHCP radiation pattern is obtained by using formula mentioned in Eqs. (6) and (7) and at last by Changing the position of test antenna orthogonal and repeat the above mention method. It has been discovered via examination of Fig. 17 that the suggested antenna possesses polarization diversity. The RHCP pattern is shown for port 1, while the LHCP pattern is shown for port 2. More than 10 dB of difference can be seen between the LHCP and the RHCP for both port 1 and port 2. It is possible to compute the LHCP and RHCP radiation patterns in the XZ plane for two distinct ports operating at 3.5 GHz by making use of the formula that is mentioned further down^41^.

6 $$ {\text{E}}_{{{\text{RHCP}}}} = \frac{1}{\sqrt 2 }\left( {{\text{E}}_{{\text{H}}} + {\text{jE}}_{{\text{V}}} } \right) $$

7 $$ {\text{E}}_{{{\text{LHCP}}}} = \frac{1}{\sqrt 2 }\left( {{\text{E}}_{{\text{H}}} - {\text{jE}}_{{\text{V}}} } \right) $$

### Diversity performance parameters

The different diversity performance parameters like envelope correlation coefficient (ECC), diversity gain (DG), channel capacity loss (CCL), mean effective gain (MEG) and total active reflection coefficients (TARC) are studied, investigated and calculated to understand the independency of each port and multi-channel propagation performance of the proposed MIMO antenna.

#### Envelope correlation coefficient and diversity gain

The diversity parameter that represents the correlation between nearby MIMO antenna components is referred to as the envelope correlation coefficient or ECC. Radiation patterns or S-parameters may be used to derive the formula for its calculation. The ECC value that is calculated by utilizing the far field radiation pattern is the one that is most highly recommended. This is because the ECC describes how the radiation patterns of different radiating components in MIMO systems are independent of one another. In addition, it can be seen that the majority of planar antennas experience loss; hence, the approach for estimating ECC that makes use of the S-parameters has to be avoided. However, if the radiation efficiency of the MIMO antenna is more than 90% then the scattering parameters-based formula can also be used to estimate the ECC values^42^. Equations (8) and (9) represent the mathematical equation for ECC, which can be obtained by using the scattering parameters and radiation pattern data of MIMO architecture respectively.

8 $$ ECC_{s} = \left| {\frac{{\left| {S_{11}^{ * } S_{12} + S_{21}^{ * } S_{22} } \right|}}{{\left| {\left( {1 - \left| {S_{11} } \right|^{2} - \left| {S_{21} } \right|^{2} } \right)\left( {1 - \left| {S_{22} } \right|^{2} - \left| {S_{12} } \right|^{2} } \right)} \right|^{1/2} }}} \right|^{2} $$

9 $$ ECC_{Far - field} = \frac{{\left| {\iint\limits_{4\pi } {\left[ {E_{i} \left( {\theta ,\phi } \right) * E_{j} \left( {\theta ,\phi } \right)} \right]d\Omega }} \right|^{2} }}{{\iint\limits_{4\pi } {\left| {E_{i} \left( {\theta ,\phi } \right)} \right|^{2} d\Omega \iint\limits_{4\pi } {\left| {E_{j} \left( {\theta ,\phi } \right)} \right|^{2} d\Omega }}}} $$

**Figure 18:**
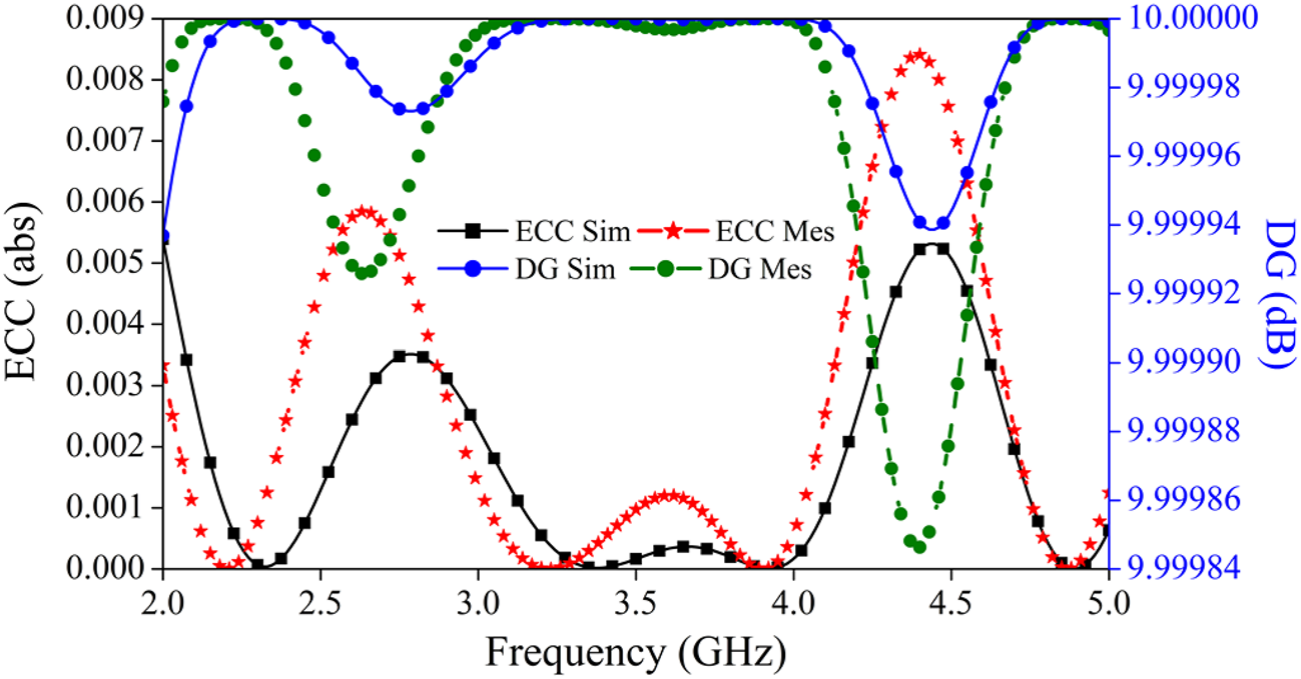
ECC and DG plot of the proposed MIMO antenna.

The simulated and calculated ECC values for the proposed radiator are shown in Fig. 18. From the perusal of Fig. 18, it is clear that the value of ECC is less than 0.005 which is far less than the acceptable limit of ECC (< 0.5)^43^.

In wireless communication systems, the quality and dependability of a MIMO antenna may be determined by its diversity gain. Therefore, the DG of the MIMO antenna has to be high (at least 10 dB) within the frequency region that is permitted. The DG is determined by using the ECC value in a calculation, and the resulting answer may be found in Eq. (10)^44,45^.

10 $$ {\text{DG}} = 10\sqrt {1 - {\text{ECC}}^{2} } $$

From the observation of Fig. 18 the value of DG for the proposed MIMO is found to be > 9.99 dB for both simulated and calculated values, which is very near to the permissible limit.

#### Channel capacity loss and mean effective gain

The CCL is the upper bound up to which the data may be carried across the communication channel with nearly no loss at all. It is defined as this maximum limit. The CCL value for a particular MIMO system is configured to be < 0.5 bits/s/Hz by default^46^. Equation (11), which uses the S-parameters as input, provides the equation for CCL.

11 $$ \begin{aligned} & CCL = - \log_{2} \det \left( {\begin{array}{*{20}c} {a_{11} } & {a_{12} } \\ {a_{21} } & {a_{22} } \\ \end{array} } \right) \\ & a_{11} = 1 - \left( {\left| {S_{11} } \right|^{2} + \left| {S_{12} } \right|^{2} } \right) \\ & a_{22} = 1 - \left( {\left| {S_{22} } \right|^{2} + \left| {S_{21} } \right|^{2} } \right) \\ & a_{12} = - \left( {S_{11}^{*} S_{12} + S_{21}^{*} S_{12} } \right) \\ & a_{21} = - \left( {S_{22}^{*} S_{21} + S_{12}^{*} S_{21} } \right) \\ \end{aligned} $$

**Figure 19:**
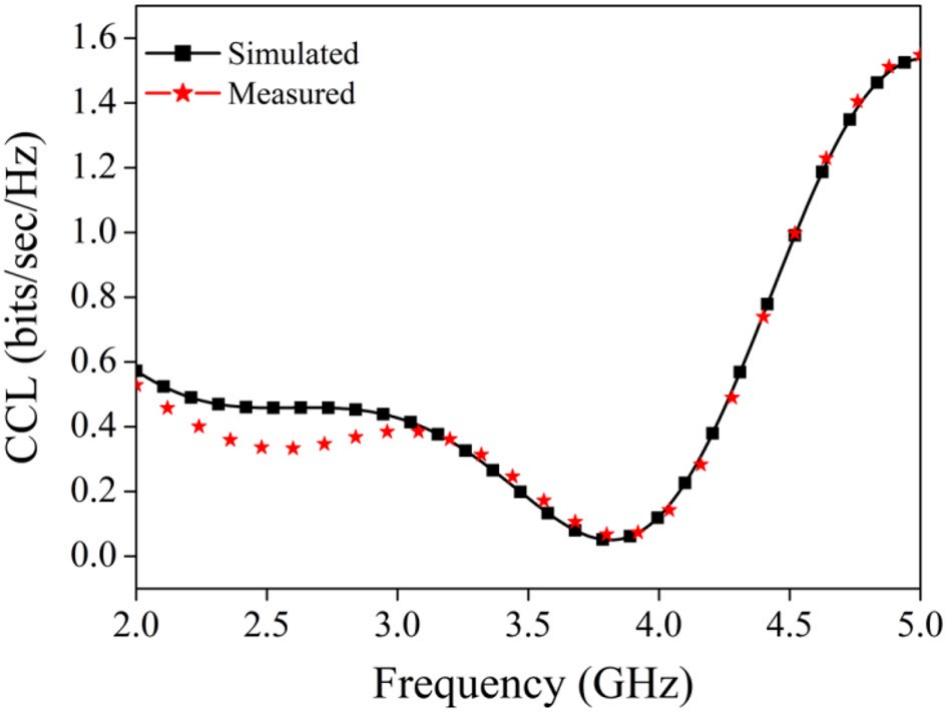
Channel capacity loss plot.

Figure 19 is representing the plot for CCL, from the perusal of Fig. 19, it is clear that the simulated and measured value of CCL is well within the permissible limit.

The MIMO antenna’s mean effective gain (MEG) is defined as the ratio of the MIMO antenna's received power to the isotropic antenna’s received power, making it a significant diversity metric for MIMO antennas. A MIMO antenna’s performance improves when the ratio of MEG_1_/MEG_2_, is less than 3 dB. Equations (12) and (13) may be used to calculate the MEG’s value^47^.

12 $$ MEG_{1} = 0.5\left[ {1 - \left| {S_{11} } \right|^{2} - \left| {S_{12} } \right|^{2} } \right] $$

13 $$ MEG_{2} = 0.5\left[ {1 - \left| {S_{12} } \right|^{2} - \left| {S_{22} } \right|^{2} } \right] $$

**Figure 20:**
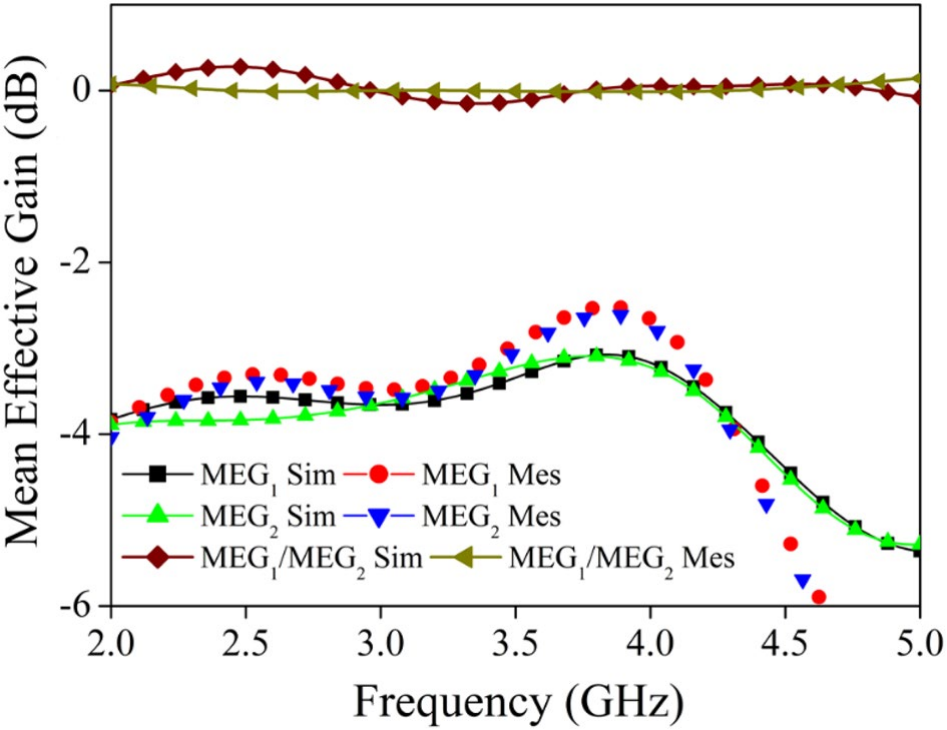
Simulated and measured MEG of the proposed MIMO antenna.

The MEG_1_, MEG_2_ and MEG_1_/MEG_2_ plot for the proposed radiator is mentioned in Fig. 20, the value of the MEG_1_/MEG_2_ is nearly equal to 0 dB.

#### Total active reflection coefficient

TARC is an additional crucial component that must be considered while determining the most efficient multiport antenna design. It illustrates the proportion of power that is reflected to the total power that is incident. For a two-port MIMO antenna the value of this parameter is determined by using the formula shown in Eq. (14)^48^.

14 $$ TARC = \frac{{\sqrt {\left( {\left( {\left| {S_{11} + S_{12} } \right|^{2} } \right) + \left( {\left| {S_{21} + S_{22} } \right|^{2} } \right)} \right)} }}{\sqrt 2 } $$

**Figure 21:**
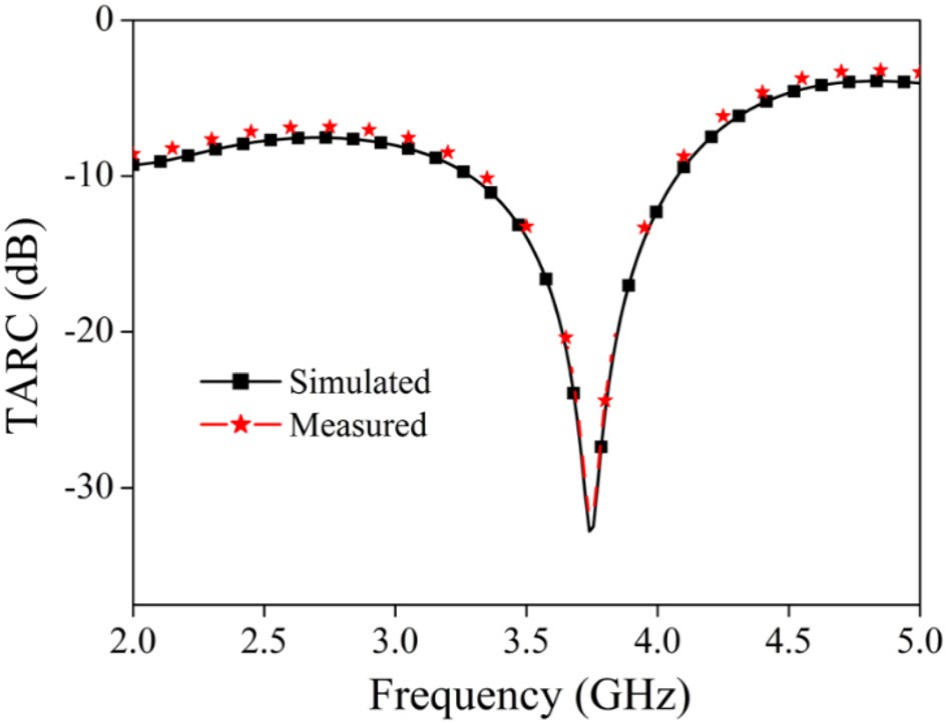
TARC plot for the designed MIMO antenna.

The reflection coefficients for ports 1 and 2 are denoted by S_11_ and S_22_, respectively. S_12_ and S_21_ stand for the isolation between the two ports. The calculated TARC of the proposed antenna is shown in Fig. 21. As improved isolation on the working band has been found between ports 1 and 2, this is indicative of well-balanced characteristics.

### Novelty justification

Table 3 provides a detailed comparison of the proposed MIMO antenna to different types of two-port radiators on the basis of bandwidth, mutual coupling between the antenna elements, electrical size (in terms of λ_0_ calculated at the lower cut-off frequency), axial ratio bandwidth, sense of polarisation and gain/radiation efficiency. According to the information that is provided in Table 3, it is clear that the suggested two-port MIMO antenna has a greater 3-dB axial ratio bandwidth when compared to other published antennas, except for reference no.^37,38^. When compared to the other described antennas in Table 3, the isolation provided by the proposed MIMO antenna is much higher. In addition, regarding the gain and radiation efficiency, the suggested model demonstrates the highest possible value, except for reference^32^. Moreover, the proposed model's diversity performance parameters are optimal in comparison to those of other MIMO antennas.

**Table 3:** Comparison of the provided two-port MIMO antenna to existing two-port antennas for Sub-6 GHz frequency of consideration.

References	Size (mm^3^) (in terms of λ_0_)/design complexity	IBW (GHz)	ARBW (GHz)	Isolation/decoupling method (dB)	Polarization	Gain (dBi)/RE (%)	ECC and DG
^49^	0.41λ_0_ × 0.33λ_0_ × 0.008 λ_0_/complex	3.3–4.2	3.2–4.25	> 15/Rectangular microstrip stub	CP	2.5/95	0.10 and 9.92
^50^	1.32λ_0_ × 1.32λ_0_ × 0.009 λ_0_/moderate	3.6–3.8	3.55–3.85	> 25/Antiparallel arrangement of the ports	CP	5/95	0.004 and NR
^51^	0.23λ_0_ × 0.52λ_0_ × 0.018 λ_0_/moderate	3.4–3.6 and 4–8	6.67–6.94	> 16/Antiparallel mirror arrangement of the ports	LP CP	3.25/75.1 3.40/74.2	0.003 and 9.95
^52^	λ_0_ × 0.48 λ_0_ × 0.0097 λ_0_/complex	3.66–3.7	3.66–3.7	> 17/Orthogonal arrangement of the ports	CP	4.6/NR	0.05 and NR
^53^	0.56λ_0_ × 0.56λ_0_ × 0.018 λ_0_/simple	3.4–3.8	NA	> 15/Orthogonal arrangement of the ports	LP	4/NR	0.09 and NR
^54^	0.375λ_0_ × 0.316λ_0_ × 0.0016λ_0_/complex	2.37–2.61, 3.30–4.41, 4.98–5.90	3.30–4.41	> 20/Antiparallel arrangement of the ports with Interlaced Lozenge Structure	CP	4/80	0.04 and 9.99
^55^	0.27 λ_0_ × 0.22 λ_0_ × 0.01 λ_0_/moderate	3.12–5	3.3–5.02	> 18.50/Parallel arrangement of the ports	CP	2/75	0.03 and NR
^56^	0.98λ_0_ × 0.73λ_0_ × 0.043 λ_0_/complex	7.90–9.59	7.90–9.59	> 18/Antiparallel arrangement of the ports with modified plus-shaped (MPS) structure	CP	3.55/70	0.01 and NR
Proposed	1.2λ_0_ × 0.6 λ_0_ × 0.008 λ_0_/simple	3–4.2	2.6–3.9	> 25/Mirror ordination of the ports	CP	4/95	0.005 and 9.99

*RE* Radiation efficiency, *LP* Linear polarization, *CP* Circular polarization, *NA* Not available, *NR* Not reported.

## Conclusion

In the presented work machine learning model-based optimized dual port MIMO antenna with RHCP/LHCP features are investigated for fifth generation new radio frequency range of n77(3300–4200 MHz) & n78 (3300–3800 MHz) band applications. Impedance bandwidth (IBW) of 3.0–4.2 GHz and 3-dB axial ratio bandwidth (ARBW) of the 2.6–3.9 GHz is reported for the proposed model. A detailed description of the CP mechanism attainment and polarization diversity features of the proposed antenna is discussed with the help of surface current distribution and radiation pattern plots. The pertinent information about the different diversity performance parameters such as ECC < 0.005, DG > 9.99, MEG_1_/MEG_2_ ≈ 0 dB, and CCL < 0.5 bits/s/Hz of the proposed MIMO antenna is incorporated in simulated and measured form. Novelty justification of the proposed design is also presented in form of a comparative investigation in terms of different vital parameters with the previously reported antennas. Three different machine learning models are used to optimize the CP in the proposed work. The machine learning-based predicted values and simulated/ measured values are found in the agreement.

## Data Availability

The datasets used and/or analysed during the current study available from the corresponding author on reasonable request.
